# Lewy body pathology is more prevalent in older individuals with mitochondrial disease than controls

**DOI:** 10.1007/s00401-019-02105-w

**Published:** 2019-11-28

**Authors:** Daniel Erskine, Amy K. Reeve, Tuomo Polvikoski, Andrew M. Schaefer, Robert W. Taylor, Nichola Z. Lax, Omar El-Agnaf, Johannes Attems, Gráinne S. Gorman, Doug M. Turnbull, Yi Shau Ng

**Affiliations:** 1 Wellcome Centre for Mitochondrial Research, Newcastle University, Newcastle upon Tyne, N2 4HH UK; 2 Translational and Clinical Research Institute, Newcastle University, Newcastle, UK; 3 NHS Highly Specialised Service for Rare Mitochondrial Disorders, Newcastle upon Tyne Hospitals NHS Foundation Trust, Newcastle upon Tyne, UK; 4 Neurological Disorders Research Centre, Qatar Biomedical Research Institute, Hamad Bin Khalifa University, P.O. Box 34110, Doha, Qatar

Mitochondrial diseases arise due to defects in mitochondrial DNA (mtDNA) or nuclear mitochondrial genes (nDNA), leading to impaired mitochondrial oxidative phosphorylation and dysfunction of organs with particularly high energy requirements. Primary mitochondrial diseases affect 1 in 4300 individuals in the UK, making them amongst the most common heritable neurological conditions [2]. Mitochondrial dysfunction has also been linked to deposition of several sporadic age-associated pathologies, including neurofibrillary tangles of hyperphosphorylated tau protein [3] and Lewy body (LB) pathology consisting of aggregated α-synuclein [4], pathological features of Alzheimer’s disease (AD) and Parkinson’s disease (PD)/dementia with Lewy bodies (DLB), respectively. Therefore, we sought to determine whether older individuals with mitochondrial diseases were at increased risk of developing age-associated neurodegenerative pathologies using post-mortem brain samples collected prospectively at Newcastle Brain Tissue Resource (NBTR).

We identified 17 cases with clinical and molecular confirmation of mitochondrial disease and sufficient tissue collected at NBTR between 2004 and 2019 (m.3243A>G *N* = 6; m.8344A>G *N* = 1; *POLGN* = 5; *SDHAN* = 1; *RRM2BN* = 1; multiple mtDNA deletions *N* = 2, single large-scale mtDNA deletion *N* = 1; Supplementary Table 1). Each case was stained with antibodies against hyperphosphorylated tau, amyloid-β and α-synuclein (Supplementary Data 1), and pathological stage was determined using international consensus guidelines [8]. We found that 5/17 mitochondrial cases (29.4%) had LB pathology, whilst a case without LB had pathology consistent with progressive supranuclear palsy (PSP; case 7). In all cases, LB pathology was labelled by antibodies against fibrillar α-synuclein and α-synuclein phosphorylated at serine 129, as in idiopathic LB disease (Fig. 1). LB pathology occurred in 4/9 (44.4%) cases with nDNA mutations, whilst it was present only in 1 case of mtDNA pathogenic variant (1/8; 12.5%). In contrast to LB pathology, amyloid-β and tau pathology were typical of the level observed in neurologically normal elderly brains and we found no evidence of age-associated pathologies such as TDP-43, age-related tau astrogliopathy and perivascular neuritic dystrophy (Supplementary Table 1). However, one case (case 11) had focal neocortical tau pathology, despite having no significant pathology in the entorhinal cortex or hippocampus (Supplementary Figure 1).

**Fig. 1:**
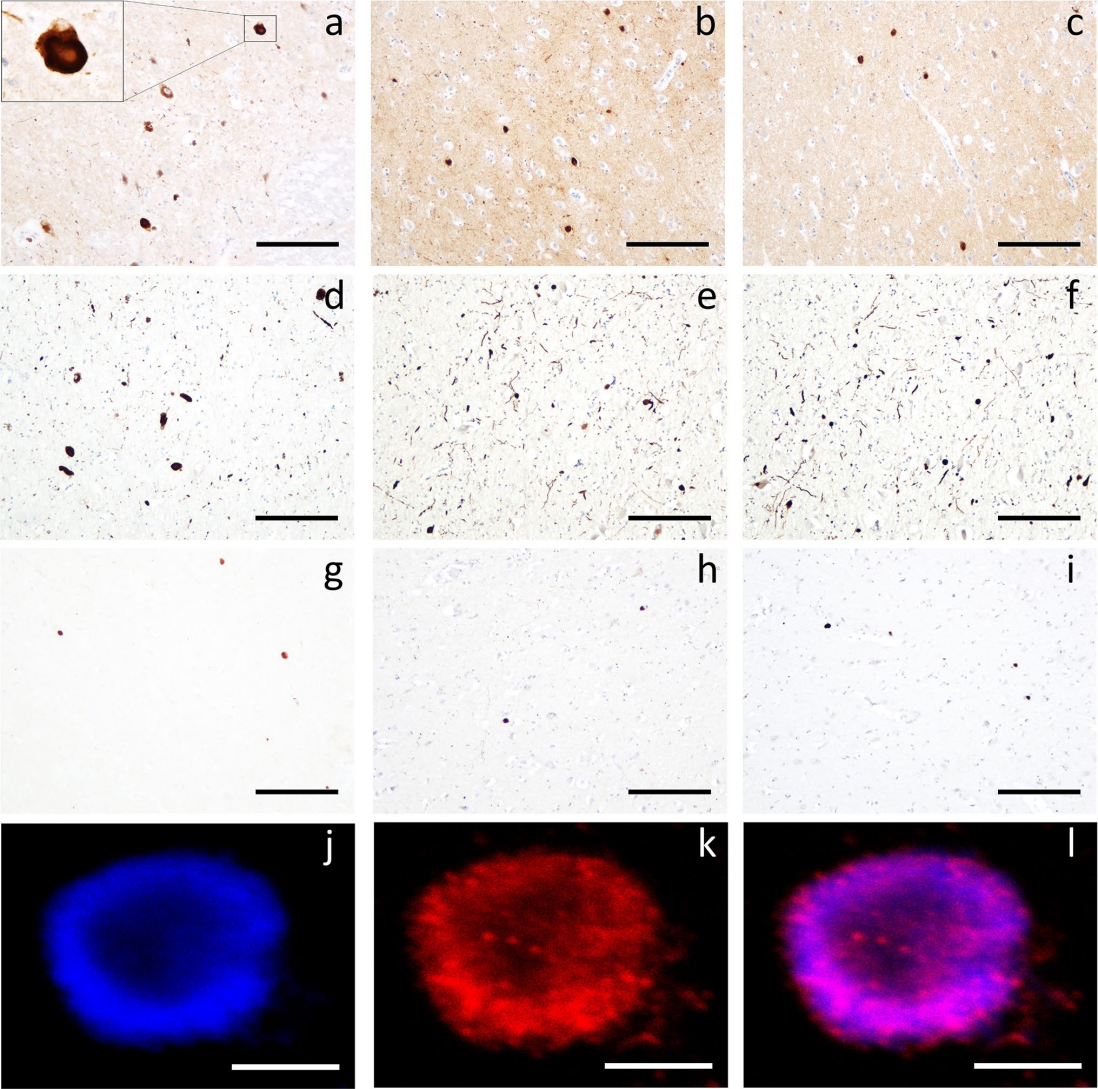
Lewy body pathology in mitochondrial disease cases. Representative examples of LB pathology in the substantia nigra (**a**), amygdala (**b**) and inferotemporal cortex (**c**) of case 2; LB pathology in the nucleus of Meynert of case 6 stained with KM51 (**d**), Syn-F2 (**e**) and pS129 (**f**); LB pathology in the amygdala of case 8 stained with KM51 (**g**), Syn-F2 (**h**) and pS129 (**i**); an LB in the substantia nigra of case 14 labelled with Syn-F2 (**j**), pS129 (**k**) and merged (**l**). Scale bars 100 µm (**a**–**i**), 5 µm (**j**–**l**)

We compared the proportion of mitochondrial cases with LB pathology to that reported in all neurologically normal control cases aged over 50 collected at NBTR since 2010, the time at which all three pathologies were systematically evaluated using the aforementioned neuropathological guidelines in control cases (*N* = 82). LB pathology was less prevalent amongst control cases (9/82, 10.9%) than mitochondrial disease cases (5/17, 29.4%; *χ*^2^ = 3.94, *p* = 0.047), despite mitochondrial cases being significantly younger (63 ± 10 years) than controls (83 ± 12 years; *p* < 0.0001). The proportion of control cases with incidental LB pathology was similar to another report of LB prevalence in control cases over 60 years old [1].

Despite the small size of this cohort, the present findings suggest LB pathology is more prevalent amongst older individuals with mitochondrial disease, particularly those with nDNA mutations, than in a comparable control population. Familial PD resulting from mitochondrial nDNA mutations does not always result in LB pathology, and the prevalence of LB pathology in *Parkin* cases is 33% based on reports from 18 cases, a comparable figure in a similarly sized cohort to our presently reported findings [6]. As mitochondrial dysfunction is associated with phenotypes of ageing [7], one could speculate that increased levels of LB pathology in this population reflect an acceleration of ageing; however, as we did not find increased levels of other age-associated pathologies, it seems unlikely that accelerated ageing alone could explain these findings. Nevertheless, it is difficult to make strong conclusions based on such a small cohort, but we hope these findings will stimulate further study of LB pathology in this population.

The clinical significance of LB pathology in this population is unclear as mitochondrial disease alone can cause significant neurodegeneration leading to parkinsonism and cognitive impairment [5] (Supplementary Data 2 and 3). We suggest the present findings highlight the need for further study of the prevalence of LB pathology in older mitochondrial disease patients. If confirmed in a larger cohort, the present findings suggest mitochondrial dysfunction and an elevated (though not absolute) risk of developing LB pathology is a feature of both aged mitochondrial disease cases and some forms of familial PD characterised by mutations in mitochondrial proteins such as *Parkin*, raising questions about the distinction between these disorders. Further understanding of LB pathology in this population is important to understand its clinical relevance in the sub-set of mitochondrial diseases cases affected by it, and to determine the role of mitochondrial dysfunction in the genesis of LB pathology.

## Electronic supplementary material

Below is the link to the electronic supplementary material.

Supplementary file1 (DOCX 2728 kb)
