# Supplementary material for: Lewy body pathology is more prevalent in older individuals with mitochondrial disease than controls
Source: Acta Neuropathol. 2019 Nov 28;139(1):219–21. doi: 10.1007/s00401-019-02105-w (PMC6942000; doi:10.1007/s00401-019-02105-w)
Supplement: Supplementary file 1 — Supplementary file1 (DOCX 2728 kb) [file 401_2019_2105_MOESM1_ESM.docx]

SUPPLEMENTARY DATA

1. Immunohistochemistry

Tissue was obtained from Newcastle Brain Tissue Resource (NBTR) following recruitment through the NHS Highly Specialised Service for Rare Mitochondrial Disorders based at Royal Victoria Infirmary, Newcastle upon Tyne, UK. We identified 18 mitochondrial disease cases aged over 50 years old that had been collected between 2004 and 2019 with available diagnostic slides stained with α-synuclein and tau, or had paraffin wax embedded tissue retained for retrospective staining and topographical evaluation. For histological evaluation, brain tissue was fixed in neutral buffered formalin, dissected, and paraffin wax embedded.

Paraffin-embedded blocks containing frontal, temporal, parietal and occipital cortices, cingulate, hippocampus, striatum, amygdala, cerebellum, midbrain and medulla were sectioned at 6 µm and stained with antibodies against hyper-phosphorylated tau (AT8; 1:4,000; Innogenetics, Ghent, Belgium), amyloid-β (4G8; 1:15,000; Signet Labs, Dedham, MA, USA), α-synuclein (KM51; 1:200; Chemicon, Hofheim, Germany). In most cases (10/17), the hippocampus was stained with TDP-43 (pS0409/410-2; 1:10,000; Cosmo Bio, Bicester, UK). Epitope unmasking was performed with heat-mediated retrieval in 0.01 mL citrate pH6 (AT8), immersion in room temperature formic acid (4G8), or heat-mediated retrieval in 1 mM EDTA (TDP-43) or 1 mM EDTA followed by immersion in room temperature formic acid (KM51). Immunopositivity was visualised using Menarini MenaPath X-cell Plus HRP detection kits (Menarini Diagnostics, Berkshire, UK), according to manufacturers’ instructions, and were counterstained with haematoxylin.

Cases that were immunopositive for LB pathology were also stained with antibodies against fibrillar α-synuclein (Syn-F2; 1:5,000; El-Agnaf laboratory, Qatar Biomedical Research Institute [12]) and α-synuclein phosphorylated at serine 129 (EP1536Y; 1:500; Abcam, Cambridge, UK). In both cases, heat-mediated antigen retrieval used 0.01 mL citrate pH6, antibody labelling was visualised with Menarini MenaPath X-cell Plus HRP detection kits as before, and counterstained with haematoxylin. Immunolabelling was visualised using a Nikon 90i microscope with a DsFi1 camera (Nikon, Tokyo, Japan).

Immunofluorescence was conducted similarly, but sections were blocked in 10% normal goat serum prior to application of primary antiserum and used appropriate secondary antibodies (goat anti-mouse IgG1 Alexa Fluor 647, goat anti-rabbit IgG Alexa Fluor 546, Thermo Fisher, 1:100) for one hour at room temperature. Autofluorescence was attenuated with incubation in Sudan Black B solution for 10 minutes at room temperature. Sections were visualised using a Leica SP8 confocal microscope (Leica, Wetzlar, Germany).

1. Description of Lewy body pathology in positive cases and comparison to mitochondrial disease neuropathology

*Case 1*

This case had moderate to severe LB pathology in the substantia nigra and nucleus of Meynert, with relatively mild pathology in other brainstem and limbic regions, and in the dorsal motor nucleus of the vagal nerve. In the cortex, only the frontal cortex showed sparse LB pathology. LBs and neurites were observed without unusual features, such as astrocytic α-synucleinopathy. Notably, frontal cortex had a micro-cystic infarct, though the few LBs in this region were not proximal to this lesion. The caudate nucleus also had a micro-infarct, and axonal spheroids containing α-synuclein were observed in this area.

*Case 2*

This case had moderate to very severe LB pathology in the dorsal motor nucleus of the vagal nerve, substantia nigra and nucleus of Meynert, with moderate changes also in limbic regions and sparse neocortical pathology in all but the occipital cortex. Frontal and parietal cortices, both of which evidenced sparse LB pathology, also had micro-infarcts but these were not unusually proximal to each other. Mineralisation of pallidal vessel walls was also observed, and the putamen and pallidum both evidenced axonal spheroids that were labelled by α-synuclein.

*Case 6*

This case had severe to very severe LB pathology in the substantia nigra, cingulate, amygdala, entorhinal cortex and nucleus of Meynert, with moderate changes to all cortical regions apart from parietal cortex, which had only sparse pathology. Mineralisation of pallidal vessels was also observed but were not proximal to any α-synuclein immunoreactive objects.

*Case 8*

This case had LBs restricted to the amygdala, where they were sparsely distributed. There were no neuropathological features associated with mitochondrial disease in the amygdala.

*Case 14*

This case had sparse to moderate LB pathology in the amygdala and nucleus of Meynert, and sparse pathology in the substantia nigra and dorsal motor nucleus of the vagal nerve. Sparse neurites were also observed in the caudate and putamen, regions that also had micro-infarcts.

*Summary*

LB pathology in mitochondrial disease patients appeared to be similar to that observed in idiopathic LB disease cases and was labelled by antibodies against fibrillar α-synuclein and α-synuclein phosphorylated at serine 129. In every case except that with LBs confined to the amygdala, the nucleus of Meynert was consistently affected most severely of all brain regions. There was no obvious association between LB pathology and the presence of focal cortical necrosis, basal ganglia mineralisations or any other neuropathological hallmarks of mitochondrial disease.

1. The relationship between clinical features of mitochondrial disease and LB disease in cases with LB pathology

Mitochondrial diseases are clinically heterogeneous, even in cases harbouring the same mutation, making it difficult to determine whether symptoms apparent *intra vitam* were related to mitochondrial disease or LB disease [5]. Nevertheless, we have discussed the potential relationships between neuropathological features observed in these patients and the underlying neuropathology.

Despite epilepsy being a common feature of many mitochondrial diseases [9], none of the cases with LB pathology exhibited epileptic symptoms, including those with *POLG* mutations. One could speculate that the lack of epilepsy may be related to the presence of LB pathology in these patients. However, it is also notable that most cases with mtDNA mutations and epilepsy had mitochondrial encephalomyopathy, lactic acidosis and stroke-like symptoms (MELAS), a condition in which epilepsy is a common feature. In contrast, the cases with nDNA mutations, including *POLG*, were relatively old, and epilepsy and stroke-like episodes in this population are very severe and often fatal in childhood or young adulthood. Therefore, the lack of epilepsy in these patients may underlie their relatively long life, enabling them to develop age-related neurodegenerative pathologies, such as LBs, rather than LB pathology having a protective effect against ictal activity.

Ataxia was observed in almost all mitochondrial disease cases with LB pathology (4/5) and cerebellar changes, particularly Purkinje cell degeneration, was observed in every mitochondrial disease case (Table 1), and we have previously reported profound respiratory chain deficits in the cerebellum of mitochondrial disease patients *post-mortem*. Due to samples of peripheral nerves not being routinely taken from patients in our clinic, we cannot exclude the possibility that peripheral α-synucleinopathy may have contributed to ataxia in these patients. However, we suggest that as cerebellar changes are well-described in mitochondrial disease [4, 6] and are a uniquely consistent neuropathological feature, it is more likely these symptoms are related to changes resulting from mitochondrial disease.

We also noted that parkinsonism was observed in 2/3 mitochondrial disease cases with LB pathology and LB Braak stages higher than 4, the stage at which LB pathology is posited to be symptomatic [2]. Parkinsonism is a central feature of LB diseases [7] and a recognised feature in some forms of mitochondrial disease, particularly those characterised by *POLG* mutations [3, 8, 11]. Furthermore, respiratory chain complex I deficiency and multiple mtDNA mutations are observed in substantia nigra neurons in mitochondrial disease, though not all patients develop extrapyramidal symptoms [11], so it is difficult to determine whether the extrapyramidal symptoms in mitochondrial disease patients in the present study reflect mitochondrial disease processes, LB disease, or both. Nevertheless, it seems likely that the clinical presentation of Patient 6, who had visual hallucinations that preceded parkinsonism by several years, was consistent with the dementia with Lewy bodies clinical phenotype.

SUPPLEMENTARY FIGURES AND TABLES


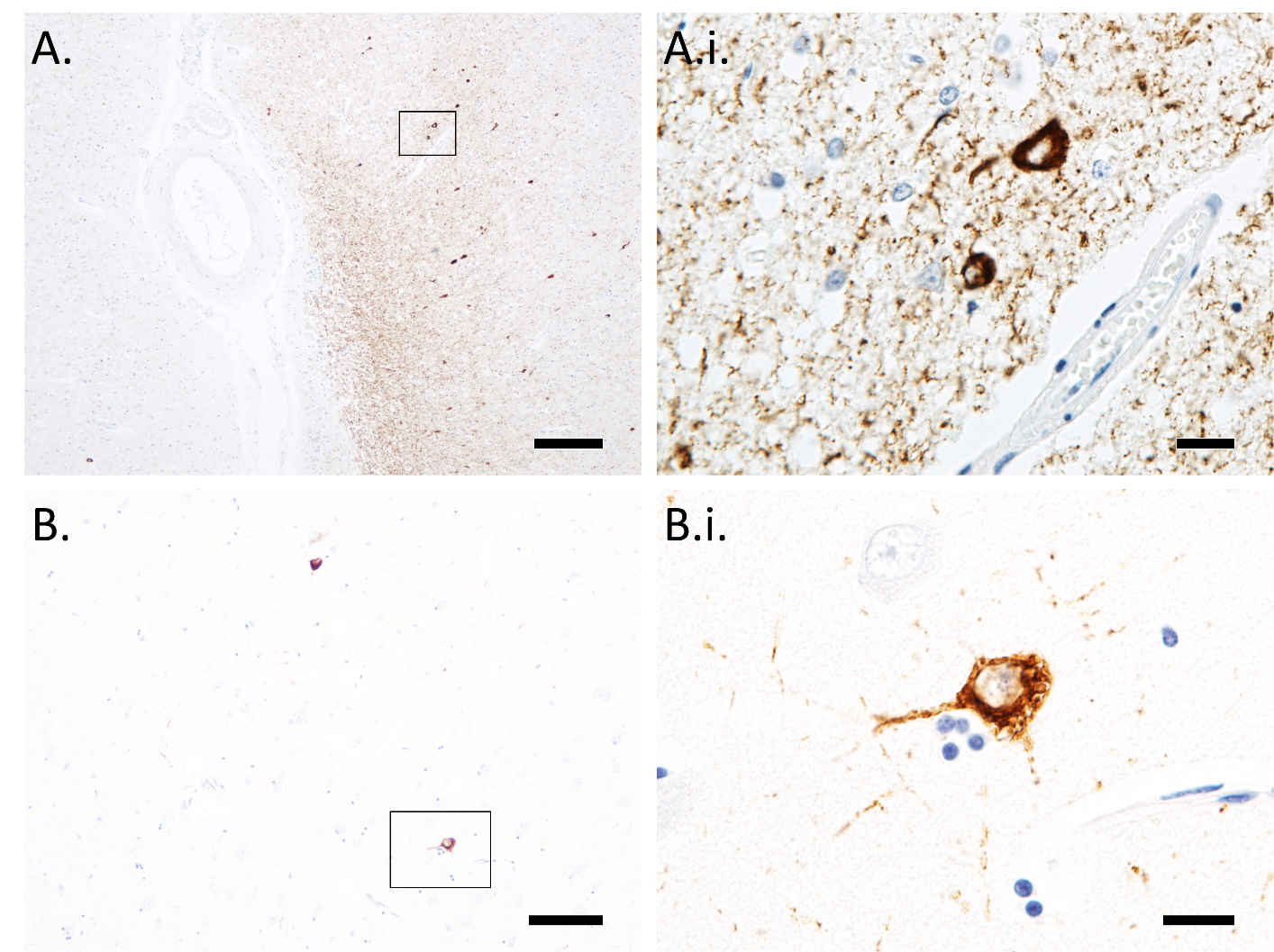


*Supplementary figure 1: Cortical tau pathology in the absence of significant hippocampal pathology. Focally severe tau pathology was observed in the gyrus of one case (A & A.i; Case 11), despite only sparse neurofibrillary tangles in the entorhinal cortex of this case (B & B.i). Scale bars = 200 µm (A), 20 µm (A.i), 100 µm (B), 10 µm (B.i).*

Supplementary table 1: Demographic details of the mitochondrial disease cohort employed in the present study. “Thal phase” refers to amyloid-β phase [10], “Braak NFT stage” refers to tau neurofibrillary pathology stage [1], and “Braak Lewy stage” refers to Lewy body pathology stage [2]. “McKeith likelihood of dementia caused by DLB” refers to the likelihood that the LB pathology is responsible for clinical dementia as described by McKeith and colleagues [7] “ad” is autosomal dominant; “ar” is autosomal recessive; “CPEO” is chronic progressive external ophthalmoplegia; “LD” is learning difficulties; “MELAS” is mitochondrial encephalopathy with lactic acidosis and stroke-like episodes; “MERRF” is myoclonic epilepsy with ragged red fibres; “mtDNA” is mitochondrial DNA; “SANDO” is sensory ataxia, neuropathy, dysarthria and opthalmoparesis. *tested negative for mutations *POLG*, *POLG2*, *ANT1, TWNK* and *RRM2B.*

| Case | Age at death | Sex | Genetic defect | | Diagnosis | Brain weight (g) | Thal phase | | Braak NFT stage | Braak Lewy stage | 4R Tau  Y/N | TDP-43  Y/N | | Mitochondrial disease neuropathology | | | | McKeith likelihood of dementia caused by DLB | |  |  |
| --- | --- | --- | --- | --- | --- | --- | --- | --- | --- | --- | --- | --- | --- | --- | --- | --- | --- | --- | --- | --- | --- |
| ***nDNA mutations*** |  |  |  | |  |  |  | |  |  |  |  | |  | | | |  | |  |  |
| Case 1 | 59 | M | *POLG* (ar) | | Parkinsonism, CPEO, peripheral neuropathy, ataxia | 1480 | N.D. | | I | 4 | N | N.D. | | Microinfarcts in frontal cortex, caudate nucleus and cerebellum; focal loss of cerebellar Purkinje cells. | | | |  | |  |  |
| Case 2 | 79 | M | *POLG* (ar) | | CPEO, ataxia, cognitive impairment | 1421 | N.D. | | IV | 5 | N | N | | Infarcts in frontal and parietal cortex, and globus pallidus; mineralisation of vessel walls in globus pallidus; cortical white matter leukoaraiosis; focal loss of cerebellar Purkinje cells. | | | | Intermediate | |  |  |
| Case 3 | 55 | M | *POLG* (ar) | | Epilepsy, cognitive impairment, ataxia, myoclonus, CPEO, peripheral neuropathy, mild LD, tremor | N.D. | 1 | | 0 | 0 | N | N.D. | | Microinfarcts in temporal cortex; loss of dentate nucleus neurons; focal loss of cerebellar Purkinje cells; Purkinje cell torpedoes. | | | |  | |  |  |
| Case 4 | 74 | F | *POLG* (ar) | | CPEO, ataxia, peripheral neuropathy | 1264 | N.D. | | 0 | 0 | N | N | | Granule cell dispersion; mineralisation of vessel walls in globus pallidus; focal loss of cerebellar Purkinje cells. | | | |  | |  |  |
| Case 5 | 52 | M | *POLG* (ar) | | SANDO, dystonia, parkinsonism, dysphagia, nocturnal hypoventilation | 1236 | 2 | | I | 0 | N | N | | Severe cortical gliosis; | | | |  | |  |  |
| Case 6 | 76 | F | *SDHA* (ad) | | Parkinsonism, restless leg syndrome, visual hallucinations (diagnosed as Charles Bonnet syndrome), ataxia, bilateral optic atrophy | 1188 | 3 | | II | 6 | N | N | | Microinfarct in occipital cortex; atrophy of occipital cortex; mineralisation of vessel walls in globus pallidus; focal loss of cerebellar Purkinje cells; Purkinje cell torpedoes. | | | | High | |  |  |
| Case 7 | 79 | F | *RRM2B* (ad) | | Indolent CPEO, rapidly progressive dementia | 1010 | 3 | | III | 0 | Y | N | | Microinfarcts in temporal and insular cortex, and cerebellum; severe hippocampal atrophy; mineralisation of vessel walls in globus pallidus. | | | |  | |  |  |
| Case 8 | 64 | F | Multiple deletions* | | CPEO, neuropathy, myopathy, ataxia | 1350 | 1 | | 0 | Amygdala only | N | N | | Microinfarct in temporal cortex; focal loss of cerebellar Purkinje cells. | | | |  | |  |  |
| Case 9 | 60 | F | Multiple deletions* | | CPEO, proximal and axial myopathy, sensory ataxia and neuropathy | 1477 | N.D. | | I | 0 | N | N.D. | | Mild microvacuolation across the cortex and in the CA1 sector of the hipopocampus. | | | |  | |  |  |
| ***mtDNA mutations*** |  |  |  | |  |  |  | |  |  |  |  | |  | | | |  | |  |  |
| Case 10 | 59 | F | m.3243A>G | | MELAS, ataxia, dementia, deafness and diabetes | N.D. | 1 | | II | 0 | N | N.D. | | Focal ischaemic lesion in CA1 sector of hippocampus; mineralisation of vessel walls of globus pallidus; focal loss of cerebellar Purkinje cells. | | | |  | |  |  |
| Case 11 | 64 | F | m.3243A>G | | MELAS; epilepsy, cognitive impairment, ataxia, deafness and diabetes | 803 | 0 | | I | 0 | N | N | | Microinfarcts in temporal cortex and pons; focally heavy tau pathology proximal to temporal microinfarct; mineralisation of vessel walls in globus pallidus; focal loss of cerebellar Purkinje cells; Purkinje cell torpedoes. | | | |  | |  |  |
| Case 12 | 53 | M | m.3243A>G | | MELAS, epilepsy, cognitive impairment, gut dysmotility | 861 | 1 | | I | 0 | N | N | | Hippocampal sclerosis; mineralisation of vessel walls in globus pallidus and dentate nucleus. | | | |  | |  |  |
| Case 13 | 54 | M | m.3243A>G | | MELAS, epilepsy, cognitive impairment, gut dysmotility | 976 | N.D. | | II | 0 | N | N | | Focally severe neuronal loss in temporal, parietal and occipital cortex; severe loss of white matter in inferior temporal cortex; mineralisation of vessel walls in globus pallidus; focal loss of cerebellar Purkinje cells. | | | |  | |  |  |
| Case 14 | 61 | M | m.3243A>G | | Recurrent encephalopathy, brainstem stroke | N.D. | 2 | | I | 3 | N | N | | Microinfarcts in temporal and parietal cortex, putamen and thalamus; focal loss of cerebellar Purkinje cells; Purkinje cell torpedoes. | | | |  | |  |  |
| Case 15 | 52 | M | m.3243A>G | | MELAS, epilepsy, cognitive impairment, gut dysmotility, end stage renal failure | N.D. | 1 | | I | 0 | N | N | | Mineralisation of vessel walls in globus pallidus and putamen; focal loss of cerebellar Purkinje cells. | | | |  | |  |  |
| Case 16 | 58 | M | m.8344A>G | | MERRF/MELAS syndrome; epilepsy, cognitive impairment, ataxia, peripheral neuropathy | 1121 | 1 | | 0 | 0 | N | N.D. | | Severe cystic infarcts in temporal, parietal and occipital cortex, and putamen; dentate nucleus neuronal loss; focal loss of cerebellar Purkinje cells; Purkinje cell torpedoes; focal loss of molecular layer cells. | | | |  | |  |  |
| Case 17 | 71 | M | Single mtDNA deletion | | CPEO, myopathy | N.D. | 1 | | I | 0 | N | N | | Mineralisation of vessel walls of globus pallidus. | | | |  | |  |  |
|  | 62.9 | 59% male | |  | | |  |  | | | | |  | |  |  |  |  |  | |  |

SUPPLEMENTARY REFERENCES

1 Braak H, Alafuzoff I, Arzberger T, Kretzschmar H, Del Tredici K (2006) Staging of Alzheimer disease-associated neurofibrillary pathology using paraffin sections and immunocytochemistry. Acta Neuropathol 112: 389-404 Doi 10.1007/s00401-006-0127-z

2 Braak H, Del Tredici K, Rub U, de Vos RA, Jansen Steur EN, Braak E (2003) Staging of brain pathology related to sporadic Parkinson's disease. Neurobiol Aging 24: 197-211 Doi 10.1016/s0197-4580(02)00065-9

3 Davidzon G, Greene P, Mancuso M, Klos KJ, Ahlskog JE, Hirano M, DiMauro S (2006) Early-onset familial parkinsonism due to POLG mutations. Ann Neurol 59: 859-862 Doi 10.1002/ana.20831

4 Habek M, Barun B, Adamec I, Mitrovic Z, Ozretic D, Brinar VV (2012) Early-onset ataxia with progressive external ophthalmoplegia associated with POLG mutation: autosomal recessive mitochondrial ataxic syndrome or SANDO? Neurologist 18: 287-289 Doi 10.1097/NRL.0b013e318266f5a6

5 Lax NZ, Gorman GS, Turnbull DM (2017) Review: Central nervous system involvement in mitochondrial disease. Neuropathol Appl Neurobiol 43: 102-118 Doi 10.1111/nan.12333

6 Lax NZ, Pienaar IS, Reeve AK, Hepplewhite PD, Jaros E, Taylor RW, Kalaria RN, Turnbull DM (2012) Microangiopathy in the cerebellum of patients with mitochondrial DNA disease. Brain 135: 1736-1750 Doi 10.1093/brain/aws110

7 McKeith IG, Boeve BF, Dickson DW, Halliday G, Taylor JP, Weintraub D, Aarsland D, Galvin J, Attems J, Ballard CGet al (2017) Diagnosis and management of dementia with Lewy bodies: Fourth consensus report of the DLB Consortium. Neurology 89: 88-100 Doi 10.1212/WNL.0000000000004058

8 Ng YS, Lax NZ, Bindoff LA, Turnbull DM (2019) Mitochondrial Neurodegenerative Disorders I: Parkinsonism and Cognitive Deficits. Diagnosis and Management of Mitochondrial Disorders. Springer, City, pp 223-239

9 Rahman S (2012) Mitochondrial disease and epilepsy. Dev Med Child Neurol 54: 397-406 Doi 10.1111/j.1469-8749.2011.04214.x

10 Thal DR, Rub U, Orantes M, Braak H (2002) Phases of A beta-deposition in the human brain and its relevance for the development of AD. Neurology 58: 1791-1800 Doi 10.1212/wnl.58.12.1791

11 Tzoulis C, Tran GT, Schwarzlmuller T, Specht K, Haugarvoll K, Balafkan N, Lilleng PK, Miletic H, Biermann M, Bindoff LA (2013) Severe nigrostriatal degeneration without clinical parkinsonism in patients with polymerase gamma mutations. Brain 136: 2393-2404 Doi 10.1093/brain/awt103

12 Vaikath NN, Majbour NK, Paleologou KE, Ardah MT, van Dam E, van de Berg WD, Forrest SL, Parkkinen L, Gai WP, Hattori Net al (2015) Generation and characterization of novel conformation-specific monoclonal antibodies for alpha-synuclein pathology. Neurobiol Dis 79: 81-99 Doi 10.1016/j.nbd.2015.04.009
